# The Turkey Ig-Like Receptor Family: Identification, Expression and Function

**DOI:** 10.1371/journal.pone.0059577

**Published:** 2013-03-18

**Authors:** Katharina Windau, Birgit C. Viertlboeck, Thomas W. Göbel

**Affiliations:** Institute for Animal Physiology, Department of Veterinary Sciences, University of Munich, Munich, Germany; University of California, Davis, United States of America

## Abstract

The chicken leukocyte receptor complex located on microchromosome 31 encodes the chicken Ig-like receptors (CHIR), a vastly expanded gene family which can be further divided into three subgroups: activating CHIR-A, bifunctional CHIR-AB and inhibitory CHIR-B. Here, we investigated the presence of CHIR homologues in other bird species. The available genome databases of turkey, duck and zebra finch were screened with different strategies including BLAST searches employing various CHIR sequences, and keyword searches. We could not identify CHIR homologues in the distantly related zebra finch and duck, however, several partial and complete sequences of CHIR homologues were identified on chromosome 3 of the turkey genome. They were designated as turkey Ig-like receptors (TILR). Using cDNA derived from turkey blood and spleen RNA, six full length TILR could be amplified and further divided according to the typical sequence features into one activating TILR-A, one inhibitory TILR-B and four bifunctional TILR-AB. Since the TILR-AB sequences all displayed the critical residues shown to be involved in binding to IgY, we next confirmed the IgY binding using a soluble TILR-AB1-huIg fusion protein. This fusion protein reacted with IgY derived from various gallinaceous birds, but not with IgY from other bird species. Finally, we tested various mab directed against CHIR for their crossreactivity with either turkey or duck leukocytes. Whereas no staining was detectable with duck cells, the CHIR-AB1 specific mab 8D12 and the CHIR-A2 specific mab 13E2 both reacted with a leukocyte subpopulation that was further identified as thrombocytes by double immunofluorescence employing B-cell, T-cell and thrombocyte specific reagents. In summary, although the turkey harbors similar LRC genes as the chicken, their distribution seems to be distinct with predominance on thrombocytes rather than lymphocytes.

## Introduction

The leukocyte receptor complex (LRC) is located on human chromosome 19q13.4 spanning about one Mb and it contains more than 40 genes including both multigene families like human killer cell Ig-like receptors (KIR) or leukocyte Ig-like receptors (LILR) as well as single copy genes like NKp46 or FCAR [1], [2]. Comparative analyses of the LRC between different mammals have revealed an extraordinary flexibility concerning gene number and haplotypes [3], [4]. For instance, the KIR gene family has been vastly expanded in man, but mice lack KIR within the LRC; instead, two KIR-like genes have been found on chromosome X [5]. Apart from mammals, gene families that are similar to the LRC encoded genes have been identified in zebra fish and channel catfish named leukocyte immune-type receptors (LITR), as well as so called novel immune-type receptors (NITR) in zebra fish and bony fish [6]–[8].

The chicken LRC is located on microchromosome 31 and encodes a single gene family designated chicken Ig-like receptor (CHIR) genes [9]–[11]. CHIR are a vastly expanded, highly diversified and polymorphic multigene family with more than 100 expressed genes in a single animal [12], [13]. They are classified as type I transmembrane proteins with either one or two C2-type Ig-like domains and are further divided into subgroups of activating CHIR-A, inhibitory CHIR-B and bifunctional CHIR-AB [10].

Activating receptors typically possess a short cytoplasmic tail without any signalling motifs, but a positively charged residue in the transmembrane domain [10], [12]. This charged residue allows the receptor to associate with an adaptor molecule like FcεRIγ mediating activation via an immunoreceptor tyrosine-based activating motif (ITAM) [14], [15].

In contrast, inhibitory receptors lack this charged residue in the transmembrane domain, but possess a long cytoplasmic tail containing immunoreceptor tyrosine-based inhibition motifs (ITIM). The ITIM motif consists of a six amino acid consensus motif, composed of L/VxYxxV/S/I/L, whereby x represents any amino acid. Ligation of the inhibitory receptor induces tyrosine phosphorylation, thereby creating a binding site for SH2 domain containing molecules and recruitment of phosphotyrosine phosphatases like SHIP, SHP-1 or SHP-2 [9], [16].

Bifunctional CHIR-AB proteins combine features of both, exhibiting a positively charged residue in the transmembrane domain and a long cytoplasmic tail including two ITIM. The membrane-proximal ITIM can be modified to an immune receptor tyrosine-based switch motif (ITSM), exchanging position −2 by a threonine instead of valine or leucine, or YxxM [10]. In comparison, the human KIR cluster also encodes similar activating and inhibitory receptors, as well as a unique receptor, KIR2DL4, that displays features of a bifunctional receptor [14], [17].

The function of CHIR in the chicken immune system are mostly unknown [11]. To date, only one CHIR ligand has been identified [14]. The bifunctional CHIR-AB1 binds to the Fc portion of IgY, an ancestral immunoglobulin isotype that is believed to be the precursor of mammalian IgG and IgE [18], [19]. Further analysis in various chicken lines identified almost 20 expressed CHIR-AB genes with variable binding properties to IgY ranging from undetectable to high affinity binding [20]. The binding depends on five critical amino acid residues that form a binding site as predicted by the three-dimensional structure [20], [21]. It has been further demonstrated that two CHIR-AB molecules bind a single IgY and the binding takes place at the Fcv3/Fcv4 domains, a site similar to the binding of IgA to the FCAR, but distinct from most mammalian FcγR. Hence the CHIR-AB-IgY-interaction resembles the binding pattern of FcαRI to the CH2/CH3-domain of IgA [22].

CHIR are presumably generated by “birth and death” evolution [23] frequently observed in multigene families where multiple duplications and deletions create an expanded gene family of one or few ancestral genes that maybe further shaped by a pathogen-driven selection process [24]. The location of the CHIR gene cluster on a microchromosome may have additionally favoured this process. In other bird species apart from chickens no CHIR-orthologs are known. For instance, CHIR could not be identified in ducks by several strategies including DNA crosshybridization with chicken probes and PCR approaches [25].

The recent progress regarding genomic databases of various birds has now provided new means of identifying CHIR homologues in other birds. Here we provide evidence for the conservation of prototypic CHIR in turkeys, a bird species closely related to chickens. These genes were designated turkey Ig-like receptors (TILR). In contrast, we were not able to identify CHIR homologues in more distantly related species. We further show that a subfamily of TILR also functions as a receptor for IgY, but that its expression pattern markedly differs from that in chickens.

## Materials and Methods

### Animals

Blood and spleen samples of lines Bxx and Bronce Kelly were obtained directly following the slaughtering process from healthy animals that were slaughtered for meat production (therefore no approval of an ethics committee is required). They were kindly provided by the slaughtering company “Süddeutsche Truthahn AG” (Ampfing, Germany). The blood samples were collected in heparin to prevent coagulation. The non-fertilized turkey egg for IgY isolation was a kind gift from Putenbrüterei Böcker (Wallhausen, Germany).

### Database Searches

For the identification of CHIR homologues in other bird species via databases, known CHIR sequences were used. Both NCBI blastx (http://blast.ncbi.nlm.nih.gov/Blast.cgi) browsing protein database using a translated nucleotide query and ensemble genome browser blat searches (http://www.ensembl.org/Multi/blastview) looking for CHIR homologues especially in turkey (Meleagris gallopavo, assembly Turkey_2.01) and zebra finch (Taeniopygio guttata, assembly taeGut3.2.4) were applied. For duck only data from Ensemble Pre were available (http://pre.ensembl.org/Anas_platyrhynchos/Info/Index). Furthermore the keyword search function of ensemble genome browser was used as well as EST databases.

### Cloning Procedures

A part of a turkey spleen was immediately taken in Ambion RNA*later* (Invitrogen life technologies, Darmstadt, Germany) and after storage over night at 4°C RNA was isolated by Trizol reagent (Peqlab, Erlangen, Germany). PBMC of a blood sample (Bxx line) were prepared by density centrifugation using Ficoll (Biochrom, Berlin, Germany) and subsequently RNA was isolated. Both spleen and PBMC RNA were reverse transcribed using the ThermoScipt™ RT-PCR System (Invitrogen life technologies, Darmstadt, Germany). Primers were designed according to partial sequences in the database and synthesized (Eurofins MWG Operon, Ebersberg, Germany). 5′ATGGCACCAATGGCGCTGGC-3′ was used as a common sense primer for amplifying TILR. 5′-CCTGCAGGGCTCCCATCTCT-3′ served as anti-sense primer for TILR-A, 5′-CACGGTAATTCAGTGCTCACTGTGG-3′ for TILR-AB and 5′-CGTGCCCACCTCGGCGTAGATA-3′ for TILR-B. Using Herculase II Fusion DNA-Polymerase (Agilent technologies, Waldbronn, Germany) PCR conditions were as follows: initial denaturation for 1 min at 94°C, three cycles at 94°C for 20 s, 65°C for 20 s and 68°C for 1 min. After that every three cycles the annealing temperature was decreased in steps of 1.0°C as far as the 21st cycle was finished. Then 13 cycles at 94°C for 20 s, 58°C for 20 s and 68°C for 1 min were added followed by a final extension at 68°C for 4 min. After purification with Wizard®SV Gel and PCR Clean-up system (Promega, Mannheim, Germany) the PCR product was cloned into pcR® Blunt II TOPO® vector (Invitrogen life technologies, Darmstadt, Germany). Colonies were tested by PCR and the plasmids from positive colonies were isolated using PureYield™ Plasmid Miniprep system (Promega, Mannheim, Germany) and sequenced (GATC, Konstanz, Germany). Sequence analysis and alignment were performed using NCBI database and DNASTAR Lasergene software package (Madison, USA). All new data has been deposited in GenBank, accession numbers are indicated in the figure legends.

### Generation of Soluble TILR-AB1

For the soluble form of TILR-AB1 (denoted as TILR-AB1-huIg) the two Ig domains of TILR-AB1 were amplified with the sense-primer 5′-GAATTCCTGCCCCCACCCT-3′ and the anti-sense primer 5′-GAATTCGCTCCCATGGGACCG-3′ (restriction sites underlined) encoding the beginning of the Ig1 and the end of the Ig2 domain. PCR was performed using Herculase II Fusion DNA-Polymerase (Agilent technologies, Waldbronn, Germany) with following PCR conditions: initial denaturation for 1 min at 94°C, 30 cycles at 94°C for 20 s, 65°C for 20 s and 68°C for 1 min adding a final extension at 68°C for 4 min using TILR-AB1 plasmid as template. The PCR product was cloned into pcR® Blunt II TOPO® vector (Invitrogen life technologies, Darmstadt, Germany). After sequencing (GATC, Konstanz, Germany) and digestion with EcoRI (Fermentas, St. Leon-Rot, Germany) the insert was ligated in pcDNA3.1/V5-His vector fusing the extracytoplasmic region of TILR-AB1 to the C_H_2-C_H_3 domain of human IgG1 [20].

### Stable Transfection and Purification

Human embryonic kidney 293 cells were cultivated in RPMI with 10% FBS and 1% penicillin-streptomycin added in a CO_2_ incubator at 37°C. Using Metafectene reagent (Biontex, Martinsried/Planegg, Germany) 293 cells were transfected with the TILR-AB1-huIg construct according to the manufacturer’s instructions. After 24 h 800 µg/ml G418 (AppliChem, Darmstadt, Germany) were added to the culture medium for selection of stable transfected cells and the resulting clones were screened for the expression of TILR-AB1-huIg on the cell surface via sandwich ELISA [20]. For this purpose, an ELISA plate was coated with anti-human IgG UNLB antibody (3 µg/ml, SouthernBiotech, Birmingham, USA), supernatants of the transfected clones were added and detected by goat anti-human IgG HRP (1∶4.000, SouthernBiotech, Birmingham, USA). Positive clones were selected and further expanded, and supernatants were subsequently purified using a protein A agarose column according to standard procedures.

### IgY Isolation and ELISA

Turkey IgY was isolated from yolk using standard procedures [26], chicken IgY was received from Jackson ImmunoReserach Europe Ltd (Newmarket, Suffolk, UK)**.** IgY of duck, grey parrot, pheasant, hawk and quail were kindly provided by B. Kaspers (Institute for Animal Physiology, University of Munich, Munich, Germany). ELISA plates were coated with IgY of turkey, chicken, pheasant, quail, hawk, duck and grey parrot (10 µg/ml). TILR-AB1-huIg was added (1 µg/ml) preparing a log2 dilution series using PBS-T and detected by goat anti-human IgG HRP (1∶4.000, SouthernBiotech, Birmingham, USA).

### Flow Cytometry

PBMC of a blood sample from chicken, duck and turkey were isolated by Ficoll (Biochrom, Berlin, Germany) [27]. 1×10^6^ cells were stained with the following mAb: 8D12 (CHIR-AB1, IgG2a) [14], 13E2 (CHIR-A2, IgG3, unpublished), 23C6 (αVβ3 integrin, IgG1, Serotec) [28] followed by anti-mouse Ig-FITC conjugate (SouthernBiotech, Birmingham, USA). The CHIR specific mab were produced by immunisation of mice with cells stably expressing the respective CHIR and validating reactivity on untransfected versus transfected cells as well as on CHIR-fusion proteins in ELISA. For double staining of turkey PBMC, cells were incubated with a primary mab including the mab listed above as well as CT4 (CD4, IgG1), 3–298 (CD8, IgG2b), 2G11 (MHC class II, IgG1). In all cases, subclass specific goat anti mouse IgG2a (for 8D12) or IgG3-FITC (for 13E2) in combination with goat-anti-mouse IgG1-PE (in the case of CT4, 23C6, 2G11), or IgG2b-PE (in the case of 3–298) conjugates were used (SouthernBiotech, Birmingham, USA). Measurements were performed by FACS Canto II flow cytometer using BD FACSDiva software (Becton Dickinson GmbH, Heidelberg, Germany).

## Results

### Three Prototypic Turkey Ig-like Receptors (TILR) can be Distinguished

**Figure 1:**
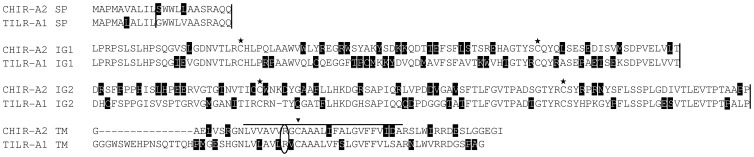
Alignment of the TILR-A sequences. The signal peptide (SP), Ig domains (IG1, IG2), transmembrane (TM) and cytoplasmic (CY) domains are indicated. Conserved cysteines forming the intrachain disulfide bridges are marked with an asterisk and the cysteine conserved in the transmembrane domain is marked by an arrowhead. The predicted transmembrane region is indicated by a line above the sequence. Note that residues that were not conserved are shaded in black. The predicted exon : intron boundaries are marked by vertical lines in the sequences. For comparison the CHIR with highest homology (CHIR-A2, accession number AJ745093) g002was used. Accession number of TILR-A1: KC201188.

**Figure 2:**
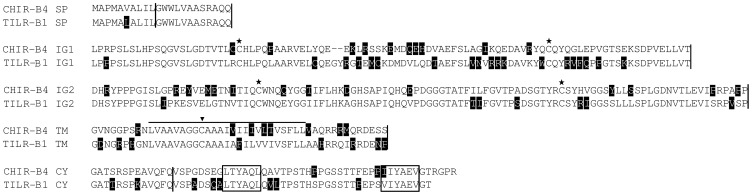
Alignment of the TILR-B sequences. The annotation of sequence features is similar to Fig. 1. ITIM sequences in the cytoplasmic domain are boxed. For comparison the CHIR with highest homology (CHIR-B4, accession number AJ639839) was used. Accession number of TILR-B1: KC201189.

**Figure 3:**
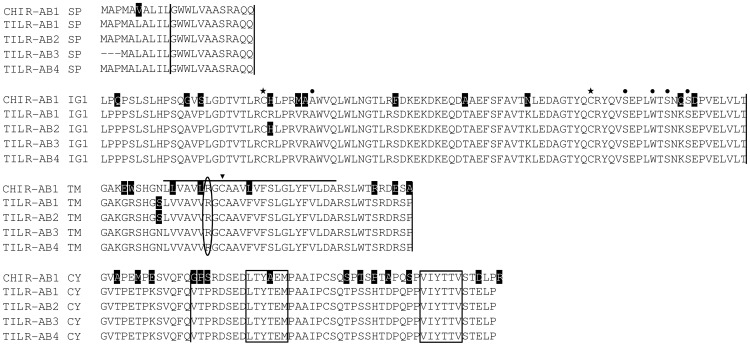
Alignment of the TILR-AB sequences. Sequence features are explained in Fig. 1. Note that TILR-AB possess a single Ig domain, only. The residues known to be important for the interaction with IgY are indicated by dots above the sequence. For comparison the CHIR with highest homology (CHIR-AB1, accession number AJ745094) was used. Accession numbers of TILR-AB1: KC201190, TILR-AB2: KC201191, TILR-AB3: KC201192, TILR-AB4: KC201193.

Using various prototypic CHIR sequences, the NCBI and ENSEMBL genome browser databases were screened for CHIR homologues in the genomes of turkey, zebra finch and duck. All our attempts to identify CHIR homologues in the latter species failed, whereas in turkey we could identify several CHIR-like fragments, but no full length genomic sequence. We therefore designed olignucleotides to amplify TILR in cDNA derived from blood or spleen. This approach yielded a total of six novel cDNA sequences that were designated “turkey Ig-like receptors” (TILR) (Fig. 1, 2, 3). In the current improved assembly of the turkey genome, additional three full length TILR sequences were annotated on chromosome 3 (accession numbers: XP_003204731, XP_003204732, XP_003204726).

All the TILR shared typical features of LRC members such as two Ig domains in the case of TILR-A and TILR-B and a single Ig domain for TILR-AB. All Ig domains were identified as C2-type Ig domains. The transmembrane regions of all TILR sequences shared a highly conserved cysteine (Fig. 1, 2, 3, marked by an arrowhead). The overall amino acid identity of 67% (CHIR-A to TILR-A), 77% (CHIR-B to TILR-B) and 84% (CHIR-AB to TILR-AB) clearly showed their relationship. The presence of full length genomic sequences in the case of TILR-A as well as the comparison to the chicken genomic sequences allowed the characterization of the genomic structures (Fig. 1, 2, 3, indicated by vertical lines). Like in other members of the LRC and in particular the CHIR sequences, the signal peptide was encoded by two exons followed by exons encoding each Ig domain. Depending on the subtype of the TILR, the connecting peptide, transmembrane region and cytoplasmic domains were either encoded by a single exon (TILR-A) or by three exons (TILR-B, TILR-AB).

The sequences could be further divided according to typical features of the transmembrane and cytoplasmic domains. The TILR-A sequences (Fig. 1) were characterized by a short cytoplasmic domain lacking any motifs and a transmembrane region containing a basic residue. These typical features potentially allow the association with adaptor molecules important for activating cellular responses. In contrast, the TILR-B sequences (Fig. 2) lacked a charged transmembrane residue, but instead displayed a long cytoplasmic domain with two ITIM, thus potentially important for inhibitory function. Finally the TILR-AB sequences (Fig. 3) combined both features of TILR-A and TILR-B in that they displayed a charged transmembrane residue and a long cytoplasmic tail with two ITIM. In conclusion, turkey TILR could be identified and categorized as TILR-A, TILR-B and TILR-AB.

### TILR-AB Binds IgY from Gallinaceous Birds

**Figure 4:**
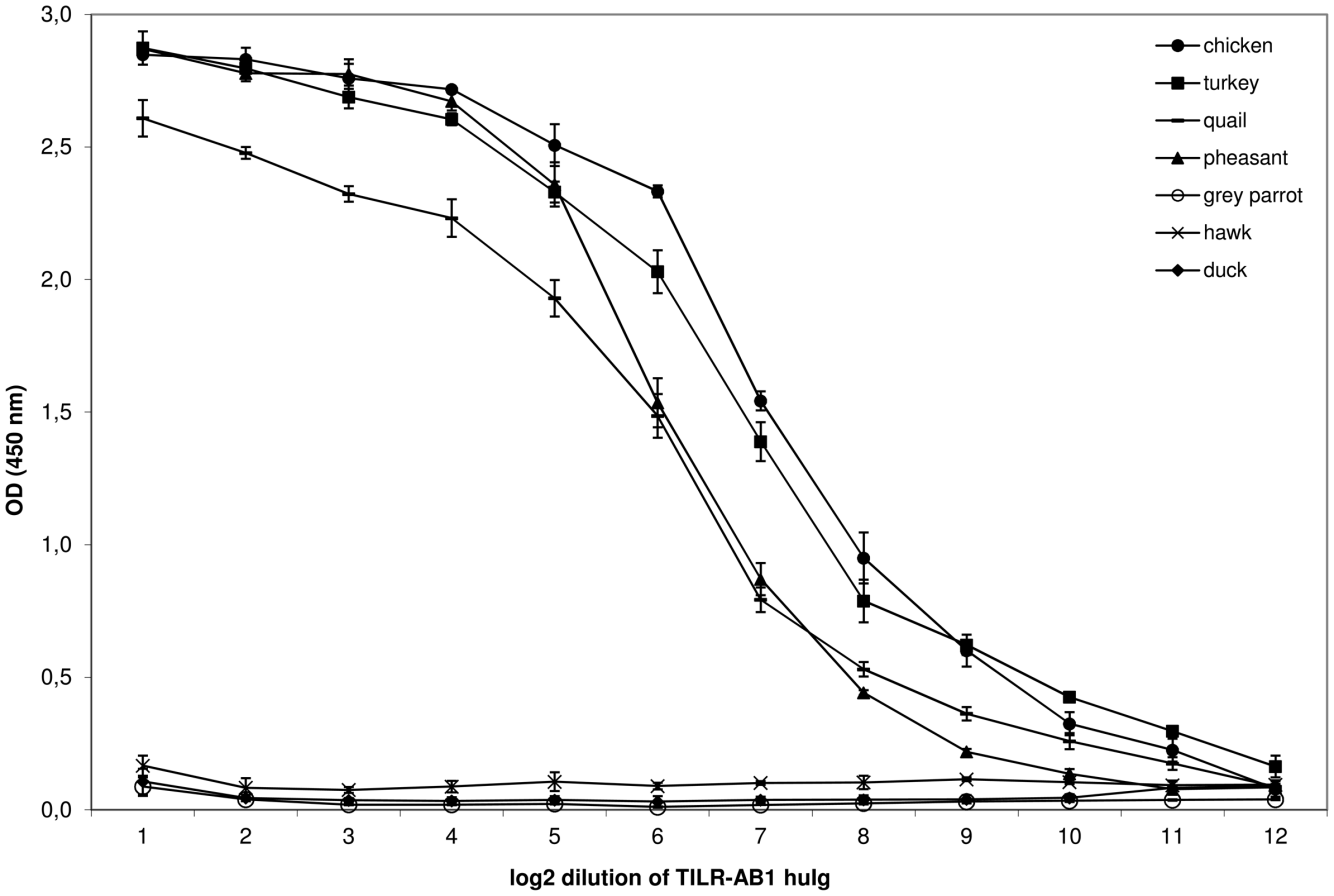
TILR-AB1 reacts with IgY from gallinaceous birds. Log_2_ dilution of TILR-AB1-huIg fusion protein on ELISA plates coated with IgY derived from birds as indicated. Mean ± SD of triplicates is shown.

To date, only IgY has been identified as ligand to interact with several CHIR-AB receptors [14]. Therefore, we also analyzed the TILR-AB sequences for the presence of those residues that have been shown to be responsible for the interaction with the CHIR-AB1 ligand, IgY [20]. All of the critical residues have been conserved in the four TILR-AB sequences (shaded in Fig. 3). These observations lead us to directly test the interaction of TILR-AB to IgY. For this purpose a human Ig fusion protein (TILR-AB1-huIg) was prepared by fusing the extracellular TILR-AB1 domain to the C_H_2 and C_H_3 domains of human IgG1. We next prepared IgY from the egg yolk of several birds, including gallinaceous as well as other bird species. The binding of the fusion protein in an ELISA revealed that the TILR-AB1-huIg fusion protein was capable of binding to IgY of all gallinaceous birds tested, including chicken, turkey, pheasant, and quail, whereas it did not react with IgY derived from parrot, duck and hawk (Fig. 4). This indicates that TILR-AB functions as a Fc receptor binding to IgY.

### CHIR Specific Mab Crossreact with TILR

**Figure 5:**
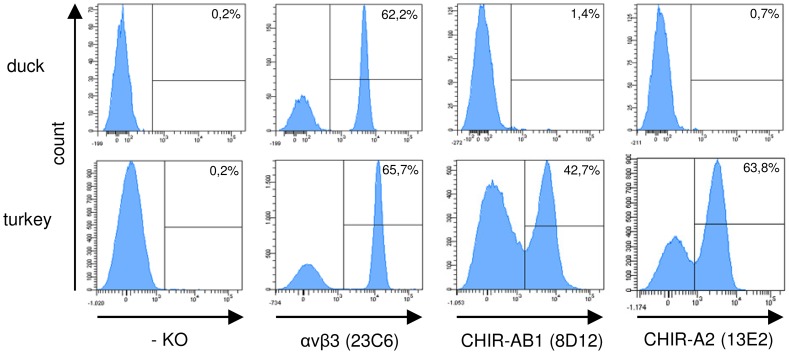
Immunofluorescence staining of duck and turkey PBMC. Blood was separated via density centrifugation and stained with mab against αVβ3 and various CHIR followed by secondary antibody conjugates. Histograms of one typical out of four separate experiments is shown and frequencies of positive cells are indicated.

The high overall homology observed between CHIR and TILR provoked us to test, whether the CHIR specific mab that we have produced against CHIR-B2, CHIR-AB1 and CHIR-A2 would crossreact with cells of duck or turkey. As a positive control for staining the αVβ3 specific mab 23C6 known to be crossreactive with many species was employed. As expected, this mab clearly reacted with a subpopulation of leukocytes in duck and turkey blood (Fig. 5). All of the CHIR specific mab were found to be unreactive with duck cells. While the CHIR-B2 specific mab did not stain turkey cells (data not shown), the CHIR-AB1 specific mab 8D12 and the CHIR-A2 specific mab 13E2 both reacted with approximately 42 to 50% and 55 to 65% of the cells depending on the animal tested, respectively (Fig. 5). Currently it is not possible to unequivocally identify the TILR which crossreact with the CHIR specific mab, however, the specificity of the 8D12 mab was confirmed by its reactivity with the TILR-AB1-huIg fusion protein (data not shown). These results further confirm the close relationship of TILR and CHIR.

### Thrombocytes and Monocytes are Reactive with CHIR Specific Mab

**Figure 6:**
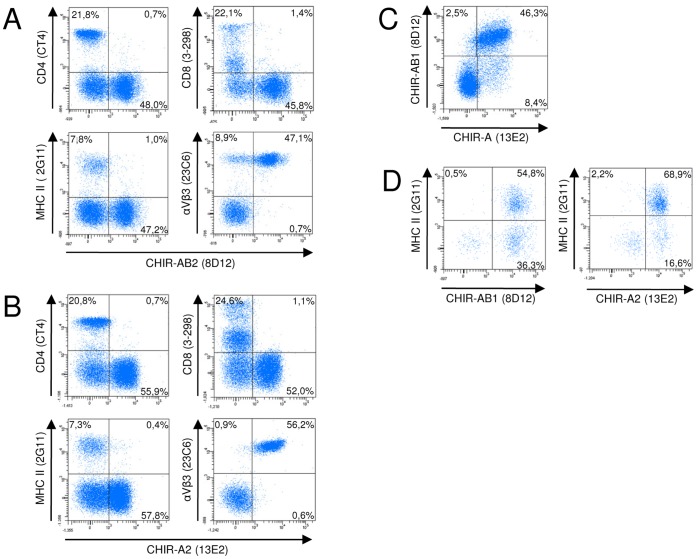
Double fluorescence analyses of turkey PBMC. Cells were labeled with either CHIR-AB1 specific mab 8D12 (A) or CHIR-A2 specific mab 13E2 (B) in combination with the T-cell specific mab CD4 and CD8, the class II specific mab 2G11 and the αVβ3 specific mab 23C6. (C) Double staining of blood leukocytes with 8D12 and 13E2. Gates were set according to forward and sideward scatter features on the lymphocyte gate in A, B and C. (D) Combined immunfluorescence of 8D12 or 13E2 in combination with the MHC class II specific mab 2G11 as in (A) or (B), but gated on the larger monocytes based on forward/side scatter characteristics. Frequencies of positive cells are indicated in the quadrants. One representative of three experiments is shown.

The finding that some CHIR specific mab crossreact with turkey cells was not anticipated, since only few of the panel of chicken leukocyte specific mab also stain turkey cells. We next wanted to characterize the cells reacting with the CHIR mab with the help of the available crossreactive leukocyte markers (Fig. 6). Here, we will designate the cells reactive with 8D12 or 13E2 as “CHIR positive” rather than “TILR positive” to stress that the mab were raised against CHIR and that the exact identity of the TILR recognized is currently unknown. Double staining with CD4 or CD8 as T cell specific reagents in conjunction with either 8D12 (Fig. 6A) or 13E2 (Fig. 6B) revealed barely any double positive cells, indicating their absence on T cells. Likewise, virtually no double positive cells were detectable in the combination of CHIR specific mab with the MHC class II mab 2G11 that served to detect turkey B cells (Fig. 6A,B). However, the αVβ3 antigen detected by the crossreactive 23C6 mab was expressed by virtually all CHIR-AB1 and CHIR-A2 positive cells in turkeys. In the case of CHIR-AB1 staining, there was also a distinct αVβ3 single positive population (Fig. 6A) that was not detectable in the CHIR-A-staining (Fig. 6B). Furthermore double immunofluorescence staining using both the 8D12 and 13E2 mab revealed that most cells coexpressed both molecules with about 2,5% and 8,4% of CHIR-A and CHIR-AB single positive cells, respectively (Fig. 6C). Since the αVβ3 integrin is mainly expressed on thrombocytes [28], we conclude that the major cell type expressing these CHIR are thrombocytes. Besides thrombocytes and lymphocytes, monocytes can be easily distinguished in PBMC preparation according to their size. When gated on the larger monocytes, double staining with the MHC class II specific 2G11 mab in combination with either of the CHIR specific mab demonstrated that the major fraction of 2G11 positive cells also coexpressed antigens recognized by the CHIR mab (Fig. 6D). In conclusion, TILR recognized by CHIR mab are mainly expressed by thrombocytes and monocytes.

## Discussion

The characterization of the TILR family extends our previous analyses regarding the chicken LRC. The assignment of the TILR as CHIR homologues is based on the high sequence homology between TILR and CHIR. Moreover, both families share a comparable structure with two C2-type Ig domains observed for the activating and inhibitory forms and a single domain present in the bifunctional receptors. Finally, they also share a common genomic structure with a typical split exon encoding the signal peptide.

CHIR are encoded by microchromosome 31, however, TILR are located on macrochromosome 3. The initial identification of TILR was hindered by the fact, that no full length sequences were found in the genomic or EST databases at that time. Consequently, we used all available information to design primers that were potentially able to amplify the three subfamilies. This has been successful, nevertheless, the number of different TILR amplified by this strategy and the comparison to the databases suggests, that the overall complexity of this family appears to be less pronounced as compared to the chicken with hundreds of CHIR found either by PCR, in EST databases or the genomic sequence. Although this observation is only preliminary and has to be substantiated by further sequencing and annotation of the respective turkey genomic region, it may point to a difference between turkeys and chickens in terms of CHIR complexity. This could also be a consequence of the different genomic location of the locus, where the position of the CHIR on a microchromosome may favor a very extensive birth and death evolution that can take place without affecting other genes located nearby, whereas this is more restricted due to the position of TILR on a macrochromosome. In this respect it is interesting to note that the position of TILR has been mapped close to the end of the macrochromosome 3. Several highly conserved genes including metastasis associated in colon cancer 1 (MACC1) and integrin 8 beta (ITGB8) have been annotated in proximity to TILR, whereas we have so far not identified unrelated genes close to the CHIR locus. Both MACC1 and ITGB8 are located on chicken chromosome 2 and thus on a different location as the CHIR present on chromosome 31. The conclusive composition of the turkey LRC remains to be characterized. In particular, it would be interesting to find out if other genes or gene families present in the mammalian extended LRC also exist in the turkey.

Our failure to find CHIR homologues in other bird species which is strengthened by a similar observation using a different approach in ducks [25] may be interpreted in different ways. In mammals the KIR locus as comparable LRC gene family has been shown to be a rapidly evolving gene family with striking differences even between closely related primates [29]. Moreover, in marine carnivores KIR are reduced to single genes [3] and other mammals like horses or rats seem to lack KIR completely [30]. A similar situation may be found in birds, where some species like chickens have a massively expanded LRC family whereas others may compensate the lack by expanding a functional homologue that does not share structural features, as is the case for Ly49 and KIR genes in mammals. It could alternatively be argued, that CHIR homologues exist in duck or zebra finch, but the strategies to identify them which were primarily based on sequence homology and some structural features were inadequate to find corresponding genes. An additional argument for the presence of at least CHIR-AB homologues would be the presence of IgY in these bird species which would argue in favor for the presence of a Fc receptor such as CHIR-AB. However, this could be questioned, since mammals lacking KIR frequently also lack the closely related and nearby located FCAR [4]. Moreover, ducks have an additional IgY-form which lacks the Fc region, so a receptor may not be necessary [18], [31], [32] and finally other Fc receptors for IgY may exist, as we have also identified an additional FcγR in the chicken [33]. The final answer whether CHIR homologues exist in bird species other than chickens and turkeys will be resolved once more bird genomes are completely sequenced and annotated.

The comparison of CHIR and TILR sequences revealed strikingly high identity between CHIR-AB and TILR-AB. This subfamily has a special function as Fc receptor binding to IgY. Turkey TILR-AB also binds to IgY and it recognizes IgY of different gallinaceous birds equally well just as we have previously shown for a CHIR-AB1 fusion protein [22]. This suggests that this CHIR/TILR-AB subfamily is primarily selected by its binding properties to the IgY Fc region and explains the high conservation of this receptor subfamily, since the Fc region of IgY is not polymorphic. Whereas the CHIR-AB sequences could be further distinguished into several subgroups that differed in their binding affinities to IgY including a subset of non-binders, that had mutations in the critical amino acid positions [20], all TILR-AB sequences identified so far, confined to the consensus that would indicate strong IgY binding. It remains to be seen, if TILR-AB sequences exist which lack binding to IgY, however, at this point the function of the non-binding CHIR-AB receptors remains to be resolved.

On the other hand the sequence comparisons of CHIR-B and CHIR-A with their corresponding TILR showed a less pronounced similarity arguing for a wider spectrum of yet undefined polymorphic ligands. As in chickens it seems plausible that the repertoire of CHIR and TILR may be shaped by yet undefined pathogens.

The finding that mab produced against several CHIR stain turkey cells was rather surprising. In general only few mab against chicken cells have been found to be crossreactive with turkey [34], [35]. Moreover, since CHIR are such a highly diverse family our attempts to make mab against particular CHIR have resulted in mab reactive with groups of highly related CHIR. The CHIR-AB specific 8D12 for instance, recognizes several CHIR-AB that bind to IgY but seems to be unreactive with non-binding CHIR-AB, in fact it is able to block IgY binding [14]. The CHIR-A specific 13E2 was made against CHIR-A2. It is able to bind several other CHIR-A, but neither CHIR-B nor CHIR-AB. So essentially, the mab recognize some members within a given subfamily, but not necessarily all. Two of the three CHIR specific mab also reacted with turkey cells. Given that the TILR and CHIR can be categorized into similar subgroups that are highly related to each other, it can be concluded that the CHIR specific mab react with similar TILR subfamily members. In the case of 8D12, this has been further documented by the reactivity of the TILR-AB1-huIg fusion protein with the 8D12 mab. The mab crossreactivity can also be explained by closest homology of TILR-A1 and TILR-AB1 to CHIR-A2 and CHIR-AB1, respectively, whereas TILR-B1 was most related to CHIR-B4 where no mab exists. All the CHIR mab tested so far in the chicken have a wide expression pattern, particularly also including lymphocytes. For instance, the CHIR-B2 specific mab is detected on virtually all lymphocytes, and CHIR-AB1 expression as detected by 8D12 can be seen on B-lymphocytes, monocytes and NK cells [9], [14]. Therefore it was quite surprising to find a distinct staining pattern in turkey cells. The main cell populations detected by both the CHIR-A2 specific 13E2 and the CHIR-AB1 specific 8D12 were thrombocytes and monocytes, whereas the mab only detected very few lymphocytes. It remains to be determined whether other TILR are expressed by lymphocytes. This striking expression difference, however, could indicate that the LRC in chicken not only evolved fast in terms of number of receptors and polymorphism, but also in functional aspects that are mirrored by the expression on different leukocyte subsets. The expression on turkey thrombocytes is of particular interest out of several reasons. Firstly, the mammalian LRC also encodes at least one receptor GPVI expressed on platelets that upon collagen binding leads to platelet activation [36]. The CHIR and TILR share the same structure and some sequence homology to GPVI, so this could indicate that some of the TILR and CHIR function as collagen receptors as has also been shown for the LRC encoded LAIR-1 [37]. Secondly, the thrombocytes, once recognized as nucleated platelets with functions limited to hemostasis, seem to have a much broader physiological function that includes important innate immune aspects. It has been demonstrated that chicken thrombocytes express some important immune receptors such as TLR and which can be triggered to secrete proinflammatory cytokines [38], [39].

Thirdly, the expression of a Fc receptor for IgY on thrombocytes suggest that thrombocytes can mediate immune functions that are dependent on antibodies. In this respect it is interesting that we have found high levels of mRNA expression of another Fc receptor, ggFcR, in thrombocytes [33]. Since the common Fcγ chain is likely expressed in thrombocytes because it is essential for receptors such as GPVI, immunocomplex binding may activate thrombocytes.

In conclusion, we have characterized homologues of the CHIR family in turkey, and in particular the FcR homologue TILR-AB that binds IgY like in chickens, but is mainly expressed on thrombocytes.
